# There is no rush to upgrade the tennis racket in young intermediate competitive players: The effects of scaling racket on serve biomechanics and performance

**DOI:** 10.3389/fpsyg.2023.1104146

**Published:** 2023-02-10

**Authors:** Pierre Touzard, Chloé Lecomte, Benoit Bideau, Richard Kulpa, Loïc Fourel, Maxime Fadier, Nicolas Cantin, Caroline Martin

**Affiliations:** ^1^M2S Laboratory, University Rennes, Rennes, France; ^2^M2S Laboratory, Inria, University Rennes, Rennes, France; ^3^Ille-et-Vilaine Tennis Departmental Committee, Maison Départementale des Sports, French Tennis Federation, Rennes, France

**Keywords:** modified sport, scaling equipment, ecological dynamic approach, children’s sport, constraints-led approach, performance analysis, injury risks, joint loadings

## Abstract

**Introduction:**

Scaling the equipment of young athletes is justified by the constraints-led approach introduced in motor learning. The aim of the present study is to analyze the effect of racket scaling on the serve biomechanics and performance parameters for young tennis players (between 8 and 11 years-old).

**Methods:**

Nine young intermediate competitive tennis players (age: 9.9 ± 1.0 years) performed maximal effort flat serves with three different rackets (scaled 23 inches, scaled 25 inches and full-size 27 inches) in a randomized order. A radar measured ball speed while shoulder and elbow kinetics and upper and lower limb kinematics were calculated with a 20-camera optical motion capture system. Repeated measures ANOVAs were used to analyze the effect of the three rackets on ball speed, percentage of serve in, serve kinematics and kinetics.

**Results:**

No significant differences in ball speed, maximal racket head velocity and percentage of serve in were observed between the three rackets. The lowest maximal upper limb kinetics and the highest upper limb maximal angular velocities were obtained with the scaled 23 inches racket.

**Discussion:**

Using scaled rackets has the advantage to decrease shoulder and elbow loadings without reducing serve performance. Consequently, the present results incite tennis coaches and parents to not upgrade too soon the size of the racket in young intermediate tennis players to avoid overuse injury risks in the long term. Our results showed that the full-size 27 inches racket induced higher lower limb kinematics. As a consequence, occasionally serving with a fullsize racket can be a sparingly interesting intervention to help young tennis players to intuitively and immediately increase their leg drive action, allowing a more functional representation of the elite junior serve.

## 1. Introduction

Improving sport performance, increasing motivation and preventing injuries constitute ones of the main concerns and responsibilities for sport coaches of young athletes. To this end, coaches are encouraged to scale the equipment in children’s sport to improve motor patterns acquisition and to favor the emergence of efficient and safe technical skills in a fun and exciting environment (Buszard et al., 2016). Scaling the equipment of young athletes is justified by the constraints-led approach introduced in motor learning (Buszard et al., 2016). This approach is based on the theory of ecological dynamics that considers humans as complex, nonlinear, neurobiological systems that interact with an unstable, unpredictable and evolving environment to produce movement patterns in a given situation (Newell, 1986; Davids et al., 2008). Three types of constraints can facilitate the emergence of self-organized movement patterns: individual characteristics, environmental and task-related constraints (Newell, 1986). In accordance with the theory of ecological dynamics in motor learning, international and national tennis organizations have proposed tennis programs with adaptations of task-related constraints in recent years [“Tennis 10s Program” (International Tennis Federation, 2012), “Galaxie Tennis” (Pestre, 2017), “Tennis Hot Shots” (McInerney et al., 2017)] based on different stages with different ball colors, racket lengths, court dimensions and net heights to facilitate the long-term technical and tactical development of tennis players. Beyond these long-term development programs, coaches can also deliberately scale equipment during training sessions to manipulate tasks constraints in the short-term hope of destabilizing the biomechanics of the existing movement pattern, encouraging exploration and self-organization towards more efficient and safer motor patterns that increase performance and reduce injury risks (Elliott et al., 2009; Reid and Giblin, 2015; Gray, 2021; Fadier et al., 2022). Among all the possible interventions on equipment, scientists encourage coaches to ask young tennis players to play with different racket size (and consequently, different mass, length, balance, swingweight and twistweight) in the theoretical hope of facilitating racket handling ability, decreasing upper limb joint loadings, promoting more variability, increasing impact performance (speed and accuracy), releasing degrees of freedom, increasing segmental and joint angular velocities (Elliott et al., 2009). Only a few of these intentions and practical interventions have been validated from a scientific point of view. Indeed, scientific studies supported the positive effects of scaled rackets on immediate tennis performance improvements. In a previous study, from 4 to 10 years old beginner children were asked to “swing as hard as possible and hit the ball as closely to the center of the racket” during a forehand hitting task with four different rackets (21 inches: 0.533 m and 0.201 kg, 23 inches: 0.584 m and 0.247 kg, 25 inches: 0.635 m and 0.293 kg, and 27 inches: 0.685 m and 0.339 kg; Gagen et al., 2005). Results showed that, for each participant, there was one specific racket that allowed better racket speed and ball impact accuracy than the other three but this “optimal” racket was not statistically related to any of the individual player’s anthropometrical data (weight, arm length, hand size, height, and leg length) or strength measures studied (shoulder strength and grip strength) or their interactions. As a consequence, the prediction of the characteristics of an optimal racket for young tennis players remains unclear.

Moreover, two other studies reported better immediate improvements in forehand performance when young beginners (6–9 years and 9–11 years) used a scaled racket (19 inches–0.483 m or 23 inches–0.584 m) with low compression balls in comparison with a standard racket (27 inches–0.685 m) and standard balls (Buszard et al., 2014a,b). However, if all these previous studies confirm that scaling racket allows to increase tennis performance in young beginners, the tennis motor skill improvements were only evaluated from a general technical point of view using video replay and technical fundamentals checklists. In these studies, biomechanical data based on full-body kinematics and upper limb joint loadings that underpin and objectify the emergence of more efficient and safer motor patterns as a consequence of the scale equipment are missing. Only two studies assessed the biomechanical effects of equipment scaling in young tennis players. In outstanding works, Buszard et al., (2020a,b) showed that a scaled racket (21 inches) and low compression balls promoted functional movement variability, whereas standard, full-size racket (27 inches) and balls resulted in the freezing of mechanical degrees of freedom during a forehand stroke task performed by beginner children (Buszard et al., 2020a). They also reported that scaled equipment promoting a more distal control of the shoulder-racket distance than full-sized equipment (Buszard et al., 2020b).

However, the biomechanical analysis of these studies was limited to the kinematics of the upper-arm, forearm and racket, but neglected the trunk, the lower limbs and the wrist. In the literature, there is also a lack of evidence that scaling equipment (i.e., tennis racket) may decrease the risk of injury by constraining children’s biomechanics to safer movement patterns inducing limited joint loadings. Moreover, all these previous studies have assessed children with limited tennis skills and experience. According to the systematic review by Buszard et al. (2016), there is a real need to examine children with a certain degree of skill regarding the task in order to explore the non-linear nature of learning within this specific population (Buszard et al., 2016).

Considered as the most important stroke in adult skilled tennis players (Johnson et al., 2006), the serve involves a proximo-distal sequence that allows the transfer of energy from the lower to the upper body (Martin et al., 2013a, 2014a) and may induce excessive joint loadings leading to overuse injuries (Martin et al., 2014b; Touzard et al., 2019). Tennis coaches spend considerable time designing specific interventions or testing different equipment constraints to improve serve biomechanics (Whiteside et al., 2014; Reid and Giblin, 2015; Fadier et al., 2022) because it is a complex and hard to control stroke, especially for young players. However, the short-term effects of the racket scaling constraint on serving performance, upper and lower body kinematics, joint loadings, and injury risks are largely unknown in young intermediate tennis players and need to be considered. As a consequence, this study aimed to assess the effect of racket scaling on the serve biomechanics and performance parameters for young intermediate tennis players. We hypothesized that racket velocity, percentage of serves in, upper limb joint kinematics to increase and upper limb joint kinetics to decrease with scaled rackets compared to full-size one. We hypothesized that scaling racket has no effect on lower limb joint kinematics and percentage of serve in.

## 2. Materials and methods

### 2.1. Participants

Nine young intermediate competitive tennis players (five boys and four girls, age: 9.9 ± 1.0 years, height: 1.39 ± 0.07 m, mass: 30.3 ± 5.1 kg), with an International Tennis Number between 6 and 9 and at least 3 years of practice, participated voluntarily in this study. Seven were right-handed and two were left-handed. All players were involved in a local training program coordinated by the Ille-et-Vilaine departmental Committee of the French Tennis Federation. At the time of testing, all the players were considered healthy, with no history of surgery on the dominant arm. Testing was conducted in an indoor tennis court at the M2S Laboratory. Before experimentation, the players and their parents provided informed consent, medical history and were fully informed about the procedures. The study was approved by the local ethics committee and conducted in accordance with the 1975 Declaration of Helsinki.

### 2.2. Experimental protocol

Forty-three retro-reflective markers were placed on the player’s bony landmarks and five markers were located on the racket as described in a previous study (Martin et al., 2014b). After a warm-up of at least 15 min, including general warm-up led by a tennis coach and serve repetitions (as many repetitions as needed to familiarize with the testing equipment), each player performed three successful flat serves (Mullineaux et al., 2001) with three different rackets (scaled 23 inches, scaled 25 inches and full-size 27 inches, respectively called R23, R25, R27) in a randomized order. The choice of these three racket sizes is in accordance with the recommendations of the national tennis federations for young tennis players between 8 and 12 years old. For each trial, two experimented tennis coaches assessed the type of serve performed and checked that the participants performed only flat serves.

For each racket condition, the players served with “green” low compression balls and from the baseline of the “green” tennis court (9.00 m from the net) to the deuce service box. The characteristics of each racket are described in Table 1. R27 corresponds to a full-size adult racket but with a weight (unstrung mass < 0.250 kg) recommended for young players who are looking for their first adult racket. The “swingweight” of each racket corresponds to the racket moment of inertia about its transverse axis. Racket moment of inertia about the long axis, called twistweight was calculated as reported in the literature (Brody, 1985):


$$\mathrm{twistweight}\;\left(\mathrm{kg}.m^{-2}\right)=\left(\mathrm{mass}\;\times \;{\mathrm{head width}}^{2}\right)/17.75$$

**Table 1:** Characteristics of the three racquets used in the study.

	R23	R25	R27
Brand and model	Wilson Burn 23	Wilson Clash 25	Wilson Six One Lite 102
Length (m)	0.584	0.635	0.685
Unstrung mass (kg)	0.190	0.239	0.249
Unstrung balance (m)	0.275	0.305	0.340
Head size (cm^2^)	613	645	658
Swingweight (kg.m^2^)	0.0159	0.0219	0.0308
Twistweight (kg.m^2^)	0.0007	0.0009	0.0010
Grip size (cm)	9.21	10.16	10.16

Players were asked to hit the ball as fast as possible and to serve into the deuce service box. A motion capture system with 20 cameras sampling at 200 Hz (Oqus, Qualisys AB., Göteborg, Sweden) was used to record the trajectories of the 3-dimensional (3-D) anatomical landmarks. Players were shirtless or wore a bra and a tight short to limit movement of the markers. Post-impact ball speed was measured for each trial with a radar (Stalker Professional Sports Radar, Applied Concepts, Plano, TX, United States) fixed on a tripod and placed 2 m behind the players in the direction of the serve. For each player, the radar’s height on the tripod was adjusted according to the impact height. After the capture, 3-D coordinates of the landmarks were reconstructed with QTM software Qualisys AB., Göteborg, Sweden with a residual error of less than 1 mm. The 3-D motions of each player were expressed in a right-handed inertial reference frame R1 whose origin was at the center of the baseline. X represented the baseline, Y pointed forward, and Z was vertical and pointed upward. The markers 3-D coordinates were filtered with a Butterworth low-pass filter with a cutoff frequency of 12 Hz as determined by residual analysis (Winter, 1990).

Different serve kinematic variables were calculated as previously described in the literature (Martin et al., 2016). An inverse dynamics approach was used to calculate maximal joint torques (Martin et al., 2014b, 2016). The serving arm was modeled as a three-link kinetic chain composed of the racket/hand segment, forearm, and upper arm. For the purpose of the study, shoulder internal rotation, abduction and horizontal abduction torques and elbow varus torque were analyzed. The joint torques obtained were first computed in the reference frame R1 and were later transformed to a series of anatomically relevant, righthanded orthogonal local reference frames at each joint. Mean kinetic peak values were normalized: torques were divided by the product of body mass by height, and then multiplied by 100 (Martin et al., 2014b).

### 2.3. Statistical analyses

For each of the three racket conditions, the magnitudes of ball speed, kinematic and kinetic parameters were averaged for each player. One-way analysis of variance with repeated measures was used to analyze the effect of the three rackets on ball speed, serve kinematics and torques. When significant main effects were present, *post hoc* pairwise comparisons were undertaken using a Holm correction to determine the source of difference. Where data were not normally distributed, significance was determined using a Friedman analysis of variance with repeated measures on ranks and a *post hoc* Durbin-Conover test. *Post-hoc* analysis with Durbin-Conover tests was conducted with a Bonferroni correction for multiple comparisons. The level of significance was set at *p* ≤ 0.05. Furthermore, we also calculated effect sizes using partial eta squared (η^2^p), defined as small (0.10–0.24), moderate (0.25–0.39), or large (≥ 0.40), Kendall’s W, defined as small (0.10–0.29), moderate (0.30–0.49) and large (≥ 0.50), and Cohen’s *r*, defined as trivial (< 0.10), small (0.10–0.30), moderate (0.31–0.50), or large (≥ 0.50) (Cohen, 1992).

## 3. Results

### 3.1. Ball speed and racket head velocity

Ball speed (*χ*^2^(2) = 3.35; *p* = 0.187; *W* = 0.186), maximal racket head velocity (*χ*^2^(2) = 2.00; *p* = 0.368; *W* = 0.111) and percentage of serve (*F*(2,18) = 0.35, *p* = 0.713, η^2^_p_ = 0.041) in were not significantly modified between the three rackets (Table 2).

**Table 2:** Serve performance for the different racket conditions (mean ± SD).

	R23	R25	R27	Main effect *p* value
Ball speed (km.h^−1^)	94.2 ± 13.7	96.2 ± 11.7	95.3 ± 8.4	0.187
Maximal racquet head velocity (km.h^−1^)	99.9 ± 13.9	98.7 ± 10.3	97.0 ± 8.2	0.368
% of serves in	49.1 ± 25.2	51.3 ± 23.8	44.2 ± 11.5	0.713

### 3.2. Shoulder and elbow kinetics

A significant and large main effect of the racket type on maximal shoulder internal rotation torque was observed (*F*(2,18) = 11.90, *p* < 0.001, η^2^_p_ = 0.598; Table 3). The maximal shoulder internal rotation torque was largely higher for R27 compared to R23 (*p* = 0.011; *r* = 0.800, large effect) and R25 (*p* = 0.005; *r* = 0.856, large effect) and for R25 compared to R23 (*p* = 0.027; *r* = 0.690, large effect). The racket type significantly and moderately influenced the shoulder abduction torque (*F*(2,18) = 4.52, *p* = 0.028, η^2^_p_ = 0.362) that is significantly higher for R27 than for R23 (*p* = 0.019; *r* = 0.722, large effect). The racket type significantly and largely influenced the maximal elbow varus torque (*χ*^2^(2) = 18.00; *p* < 0.001; *W* = 1.000) that is significantly higher for R27 than for R23 (*p* < 0.001; *r* = 0.629, large effect) and in R25 (*p* < 0.001; *r* = 0.629, large effect), and for R25 compared to R23 (*p* < 0.001; *r* = 0.630, large effect; Table 3).

**Table 3:** Maximal joint loadings for the different racket conditions (mean ± SD).

Maximal joint loadings (% BW*H)	R23	R25	R27	Main effect *p* value	*Post-hoc* difference and effect size
Shoulder internal rotation torque^***^	41.9 ± 8.3	47.5 ± 7.9	51.1 ± 8.5	< 0.001	R27 > R23 (large effect) R25 > R23 (large effect) R27 > R25 (large effect)
Shoulder abduction torque^*^	60.6 ± 24.1	69.6 ± 21.8	79.3 ± 30.9	0.028	R27 > R23 (large effect)
Shoulder horizontal abduction torque	41.9 ± 25.2	41.3 ± 17.0	48.0 ± 14.8	0.050	/
Elbow varus torque^***^	43.7 ± 8.8	49.6 ± 7.4	54.7 ± 7.9	< 0.001	R27 > R23 (large effect) R25 > R23 (large effect) R27 > R25 (large effect)

Significant main effect between racket conditions: ^*^p < 0.05; ^***^p < 0.001. BW: bodyweight, H: height.

### 3.3. Upper body kinematics

The results showed a significant and large main effect of the racket type on maximal velocity of forearm pronation (*F*(2,18) = 5.50, *p* = 0.015, η^2^_p_ = 0.407). The maximal velocity of forearm pronation was largely higher for R23 compared to R27 (*p* = 0.028; *r* = 0.688, large effect). The racket type significantly and moderately influenced the maximal velocity of elbow extension (*χ*^2^(2) = 6.89; *p* = 0.032; *W* = 0.383) that is significantly lower for R27 than for R25 (*p* = 0.007; *r* = 0.628, large effect). The racket type significantly and largely influenced the maximal velocity of wrist flexion (*χ*^2^(2) = 11.56; *p* = 0.003; *W* = 0.642) that is significantly lower for R27 than for R25 (*p* = 0.002; *r* = −0.628, large effect) and in R23 (*p* < 0.001; *r* = −0.489, moderate effect; Table 4).

**Table 4:** Maximal upper body kinematics for the different racket conditions (mean ± SD).

	R23	R25	R27	Main effect *p* value	*Post Hoc* Difference and effect size
Upper trunk longitudinal rotation velocity (°.s^−1^)	672 ± 205	675 ± 158	642 ± 151	0.522	/
Trunk flexion velocity (°.s^−1^)	263 ± 79	266 ± 59	275 ± 58	0.695	/
Pelvis longitudinal rotation velocity (°.s^−1^)	555 ± 192	567 ± 149	495 ± 207	0.717	/
Shoulder-over-shoulder rotation velocity (°.s^−1^)	255 ± 83	259 ± 65	268 ± 61	0.643	/
Shoulder internal rotation velocity (°.s^−1^)	2016 ± 725	1772 ± 600	1,656 ± 540	0.062	/
Forearm pronation velocity (°.s^−1^) *	1,644 ± 794	1,442 ± 740	1,345 ± 661	0.015	R27 < R23 (large effect)
Elbow extension velocity (°.s^−1^) *	1,126 ± 392	1,128 ± 299	1,050 ± 283	0.031	R27 < R25 (large effect)
Wrist flexion velocity (°.s^−1^) **	1748 ± 299	1,644 ± 242	1,483 ± 235	0.003	R27 < R23 (moderate effect) R27 < R25 (large effect)

Significant main effect between racket conditions: ^*^p < 0.05; ^**^p < 0.01.

### 3.4. Lower body kinematics

The racket type significantly affected the angles of maximal front knee flexion (*F*(2,16) = 7.36, *p* = 0.005, η^2^_p_ = 0.479) and back ankle flexion (*F*(2,16) = 4.27, *p* = 0.033, η^2^_p_ = 0.348). Maximal front knee flexion was significantly lower for R23 than for R25 (*p* = 0.044; *r* = 0.547, large effect) and in R27 (*p* = 0.044; *r* = 0.736, large effect; Table 2). The results showed a significant and large main effect of the racket type on maximal extension velocities of the back knee (*F*(2,16) = 8.08, *p* = 0.004, η^2^_p_ = 0.502) and the back ankle (*F*(2,16) = 6.61, *p* = 0.008, η^2^_p_ = 0.452). The maximal velocity of back knee extension was significantly lower for R23 compared to R25 (*p* = 0.032; *r* = 0.760, large effect) and R27 (*p* = 0.033; *r* = 0.729, large effect). The maximal velocity of back ankle extension was significantly lower for R23 compared to R27 (*p* = 0.047; *r* = 0.734, large effect). The racket type significantly and moderately influenced the maximal front hip vertical velocity (*χ*^2^(2) = 6.89; *p* = 0.032; *W* = 0.383) that is significantly lower for R23 than for R27 (*p* = 0.007; *r* = −0.544, large effect; Table 5).

**Table 5:** Maximal lower body kinematics for the different racket conditions (mean ± SD).

	R23	R25	R27	Main effect *p* value	*Post-hoc* Difference and effect size
Internal angle of maximal back ankle flexion (°) *	81 ± 6	79 ± 8	79 ± 7	0.033	NS
Internal angle of maximal front ankle flexion (°)	81 ± 7	79 ± 8	81 ± 8	0.052	/
Internal angle of maximal back knee flexion (°)	128 ± 15	126 ± 16	127 ± 16	0.874	/
Internal angle of maximal front knee flexion (°) **	125 ± 11	121 ± 9	120 ± 9	0.005	R27 < R23 (large effect) R25 < R23 (large effect)
Back knee extension velocity (°.s^−1^) **	343 ± 201	407 ± 205	416 ± 203	0.004	R27 > R23 (large effect) R25 > R23 (large effect)
Front knee extension velocity (°.s^−1^)	381 ± 154	421 ± 138	454 ± 111	0.059	/
Back ankle extension velocity (°.s^−1^) **	452 ± 120	503 ± 135	530 ± 110	0.008	R27 > R23 (large effect)
Front ankle extension velocity (°.s^−1^)	407 ± 148	500 ± 97	485 ± 119	0.097	/
Back hip vertical velocity (m.s^−1^)	1.3 ± 0.4	1.4 ± 0.3	1.4 ± 0.3	0.097	/
Front hip vertical velocity (m.s^−1^) *	0.9 ± 0.3	1.0 ± 0.2	1.0 ± 0.2	0.031	R27 > R23 (large effect)

Significant main effect between racket conditions: ^*^p < 0.05; ^**^p < 0.01. NS: *post-hoc* tests revealed no significant difference between the three racquet conditions.

## 4. Discussion

This study provides an evidence-based insight into the constraint-led approach by evaluating a contemporary coaching intervention and task constraint in tennis. Our results demonstrate that, at the same time, scaling tennis racket can both positively and negatively affect the joint and segment biomechanics of different body parts.

The results obtained in this study for young intermediate tennis players seem to show that scaling racket from 23 to 27 inches would not have immediate effect on performance and percentage of serves in. As expected, upper limb joint kinetics increased with the length, the mass and the moments of inertia of the racket. It appears that serving with a light full-size racket altered several upper limb kinematics but simultaneously and surprisingly improved several lower limb kinematics for young tennis players.

As expected, our results showed that when racket size, mass, swingweight and twistweight increased from R23 to R27, the maximal shoulder and elbow torques were significantly higher in the young tennis players involved in our study. Our findings are in agreement with a previous study in expert adult players who performed sets of serves using two rackets identical in mass, balance, and swingweight but with different twistweights (0.00152 vs. 0.00197 kg.m^2^; Rogowski et al., 2014). Significant increases in maximal shoulder and torques were associated with the increase in racket twistweight. These findings suggest that when racket characteristics (mass, length, swingweight and twistweight) increase, the dominant upper limb joints are required to produce more torque to swing the R27 rather than the two other rackets. Increasing maximal joint torques during the tennis serve is associated with higher risks of overuse upper limb injuries (Martin et al., 2013b, 2014b). Consequently, our results confirm our initial hypothesis as well as those of medicine practitioners and researchers, who have suggested that task-related constraints and especially a full-size racket could overload the upper limb joints (Miller, 2006; Hennig, 2007; Gray, 2021) and, potentially increase the risks of upper limb injuries in young tennis players. In a recent study, incidence proportion revealed that 13.0% of U10 and 61.0% of U12 players in a tennis academy sustained an injury over 2 years of tennis practice (O’Connor et al., 2020). In young tennis players, the shoulder and elbow showed the highest frequency in the upper limb (Kibler and Safran, 2005; O’Connor et al., 2020). The higher shoulder horizontal abduction and internal rotation torques generated from R23 to R27 may induce a greater risk of rotator cuff overuse injuries and shoulder tendinopathies that are common in young tennis players (Bylak and Hutchinson, 1998). The higher maximal elbow varus torque generated from R23 to R27 may increase the risk of elbow tension injuries, such as lateral epicondylitis (tennis elbow), medial epicondylitis, and injury to the medial epicondylar apophyseal growth plate that are common in skeletally immature tennis players (Bylak and Hutchinson, 1998).

In our study, the highest upper limb maximal angular velocities were obtained with R23. This result is in agreement with a previous work reporting that when the racket swingweight was increased for adolescent tennis players, the maximal shoulder internal rotation and wrist flexion velocities during the serve both decreased and even regressed towards the values documented for pre-pubescent players (Whiteside et al., 2014). These kinematical changes prove that the degrees of freedom are reorganized and that the movement system is constrained by equipment characteristics. The immediate decreased angular velocities were probably measured because the young intermediate tennis players involved in our study lacked the upper limb strength to overcome the higher characteristics of the full-size racket (R27). While the shoulder internal rotation, the wrist flexion and the elbow extension velocities are crucial contributors to the serve speed (Gordon and Dapena, 2006) and decreased from R23 to R27, our results showed no significant immediate difference in ball speed between R23, R25, and R27. Maximal racket head velocity ranged from 99.9 to 97.0 km.h^−1^ (−2.9 km.h^−1^) between R23 and R27, but this decrease was not statistically significant. Whiteside et al. (2014) increased the racket swingweight by 10% for a population of adolescent tennis players and found a significant but very small decrease in the resultant velocity of the racket at impact (−3.2 km.h^−1^) although not in ball speed (Whiteside et al., 2014). Creveaux et al. (2013) investigated the influence of three rackets with distinct mass, balance, swingweight, twistweight and transverse moment of inertia on serve biomechanics in adult expert players. They reported similar ball speeds for the three racket conditions (Creveaux et al., 2013). Söğüt (2017) showed that adding extra mass (+ 10 and + 20 grams) to the tip of a racket has no acute effect on serve ball speed for a population of junior tennis players (Söğüt, 2017). Based on the study of Whiteside et al. (2014), we suggest that the decreases in upper limb angular velocities observed with R27 and R25 were offset by the increase in racket mass, a more efficient impact, or both and could explain why we did not observe a significant decrease in ball speed and racket head velocity from R23 to R27.

Contrary to our hypothesis, our results showed that serving with a full-size racket (R27) promoted a more dynamic engagement of the leg drive from the players. This outcome is deduced from the increase in maximal back knee and ankle extension velocities (respectively + 73°.s^−1^ and + 78°.s^−1^ with R27 than with R23), the higher front knee flexion angle (+5° with R27 than with R23) and the higher maximal front hip vertical velocity (+0.1 m.s^−1^ with R27 compared to R23). Our results suggest that the R27 releases the degrees of freedom of the front knee. With R27, the back knee and ankle extension velocities and the front knee flexion angle were closer to previous published data obtained in older and more skilled players (elite juniors; Whiteside et al., 2013; Fett et al., 2021) and therefore point towards a more mature and efficient leg drive. Indeed, in order to hit a proficient serve, tennis players need to produce an efficient leg drive based on first an effective ankles and knees flexion and then a vigorous ankles and knees extension (Girard et al., 2007; Reid et al., 2008). The constraint-led approach stipulates that the constraints imposed by sport (e.g., court size, net height, balls and racket characteristics in tennis) both determine the boundaries of what actions are possible and provide opportunities for action called “affordances” (Newell, 1986; Davids et al., 2008). Our results support this approach because they suggest that a heavier, bigger and higher inertia racket provides an affordance to young intermediate tennis players by forcing them to intuitively increase their leg drive during serve, permitting a more functional representation of the elite junior serve. According to previous studies (Withagen et al., 2012, 2017), certain information sources (visual, acoustic, haptic and proprioceptive) in a performance environment “invite” actions. Proficient tennis players are able to distinguish differences as small as 2.5% in the swingweight of a racket (Brody, 2000). Inexperienced tennis children are also sensible to different rackets’ swing weight (Beak et al., 2000). We hypothesize that the young intermediate tennis players in our study perceived the higher mass, length and swing weight of R27 which “invited” them to produce immediate better leg drive kinematics, perhaps in an attempt to compensate for their immature upper limb musculature. By increasing the action of their leg drive, we would expect that young tennis players could be able to transfer a little bit more force to their trunk and their dominant upper limb. However, even if the leg drive kinematics were moderately to largely improved with R27, we did not see any immediate repercussions on the trunk and the dominant upper limb. Indeed, our results showed no significant differences in trunk kinematics between the three racket conditions and even highlighted a significant decrease in several upper limb angular velocities (forearm pronation, elbow extension and wrist flexion, tendency for the shoulder internal rotation) with R27. The lack of energy and force transfer between the leg drive and the trunk can be explained by the immature serve technique of the young (pre-pubescent) tennis players (Whiteside et al., 2013; Fadier et al., 2022).

### 4.1. Practical implications

Our results allow to propose practical applications for tennis coaches and parents. Using scaled rackets (R23) has the advantage to decrease shoulder and elbow loadings without reducing serve performance (ball speed and maximal racket head velocity). Consequently, the present results incite tennis coaches and parents to not upgrade too soon the size of the racket in young intermediate tennis players to avoid overuse injury risks in the long term. Our findings also encourage coaches to incorporate deliberate perturbations of the service action with different rackets to induce immediate and more appropriate coordinative joint rotations in young intermediate tennis players. Using scaled rackets (R23) has the advantage to increase the maximal angular velocities of the most distal joints and segments (forearm pronation, elbow extension and wrist flexion) that are generally restrained or sacrificed in prepubescent tennis players to simplify the serve motion. This intervention can be regular since our results showed significant decreases of shoulder and elbow loadings with this type of racket. On the contrary, serving with a full-size (R27) but light mass racket (<0.250 kg) can be an interesting intervention to help them to intuitively and immediately increase their leg drive action, allowing a more functional representation of the elite junior serve. Obviously, this intervention needs to be occasional and used with a lot of moderation and caution since our results showed significant increases of shoulder and elbow loadings with this type of racket.

### 4.2. Limitations and future directions

This study has some limitations. First, our sample size is limited because we only included young intermediate tennis players and their participation was voluntary and submitted to their parents’ consent. Some results (shoulder horizontal abduction torque, maximal shoulder internal rotation velocity, front knee and ankle extension velocities, back hip vertical velocity) tend to show differences between the three rackets. It seems reasonable to assume that nonsignificant results are due to lack of power caused by the small number of subjects involved in the study and the small number of successful serves (only three) used for the statistical analysis. One may assume that with more trials and fatigue related to differences in racket characteristics (mass and length), other significant statistical differences would appear. Similarly, given the small sample size, the current study lacks generalization and thus serves to encourage future work to examine and confirm the immediate effects of racket scaling on serve biomechanics on larger or different tennis playing cohorts. Moreover, it would be interesting to conduct future studies about the immediate effects of racket scaling on groundstrokes (forehand and backhand) kinematics and kinetics. Our study did not take into account the individual characteristics of the young players involved (age, body height, mass, upper and lower limb strength) and we did not know which scaled racket was the most appropriate for each child. Consequently, in an attempt to discover the most optimal scaling ratio, future studies should consider applying concepts from the body-scaling literature, namely pi ratios that offers a practical and seemingly new means to quantify the most beneficial scaling ratio on the basis of individual characteristics. Moreover, in the literature, it has been reported that the age and the maturation have an effect on serve biomechanics in elite female tennis players (Whiteside et al., 2013). Indeed, several racket and ball kinematics are different between elite pre-pubescent, pubescent and post-pubescent female tennis players. In our work, we did not take into account the maturity status of the players involved in the experimentation. This is a limitation of our study. To go even further, future longitudinal investigations should also examine the influence of equipment modification in relationship with the players’ maturity status and strength on serve learning over a certain period of time to better understand the motor learning process.

## 5. Conclusion

In conclusion, the current results seem to show that scaling racket from 23 to 27 inches would not have immediate effect on ball speed, maximal racket head velocity and percentage of serves in but would decrease shoulder and elbow loadings. Moreover, the manner in which the body produced joint angular velocities differed between the three racket conditions, with scaled rackets promoting more distal angular velocities and the full-size racket facilitating more proximal angular velocities from the lower limbs. Our results suggest that serving with a full-size racket provides beneficial biomechanical opportunities for the lower limbs but detrimental boundaries for the dominant upper limb in young intermediate tennis players (between 8 and 11 years old). Finally, our study shows that modifying racket characteristics constitutes a short-term and relevant practical intervention that provides immediate new learning opportunities for young intermediate tennis players.

## Data availability statement

The raw data supporting the conclusions of this article will be made available by the authors, without undue reservation.

## Ethics statement

The studies involving human participants were reviewed and approved by University of Rennes 2. Written informed consent to participate in this study was provided by the participants’ legal guardian/next of kin.

## Author contributions

CL, PT, MF, BB, RK, LF, NC, and CM: conceptualization. CL, PT, MF, NC, and CM: data curation and methodology. CL, MF, and CM: formal analysis. CL, PT, and CM: writing—original draft. PT, LF, and CM: writing—review and editing. All authors have read and agreed to the published version of the manuscript.

## Funding

This research was funded by the M2S Laboratory of Rennes 2 University.

## Conflict of interest

The authors declare that the research was conducted in the absence of any commercial or financial relationships that could be construed as a potential conflict of interest.

## Publisher’s note

All claims expressed in this article are solely those of the authors and do not necessarily represent those of their affiliated organizations, or those of the publisher, the editors and the reviewers. Any product that may be evaluated in this article, or claim that may be made by its manufacturer, is not guaranteed or endorsed by the publisher.

## Abbreviations

R23: scaled 23 inches racket.
R25: scaled 25 inches racket.
R27: full-size 27 inches racket.
